# Utilizing Audience Response to Foster Evidence-based Learning in a Pilot Study: Does It Really Work?

**DOI:** 10.7759/cureus.3799

**Published:** 2018-12-31

**Authors:** Omer Awan, Farouk Dako, Talal Akhter, Sana Hava, Faiq Shaikh, Sayed Ali, Paul Chang

**Affiliations:** 1 Radiology, University of Maryland Medical Center, Baltimore, USA; 2 Radiology, Temple University Hospital, Philadelphia, USA; 3 Radiology, Cellsight Technologies, San Francisco, USA; 4 Radiology, University of Chicago Medical Center, Chicago, USA

**Keywords:** audience response, radiology education, learning outcomes

## Abstract

Introduction

Radiology residency programs are increasingly using audience response systems (ARS) in educational lectures. It is imperative that this is investigated to assess if learning outcomes in trainees are actually improved.

Methods

The primary objective of this randomized prospective unblinded pilot study was to assess the effect of ARS on long-term learning outcomes, with a secondary objective of understanding perceptions of ARS amongst radiology residents. Twenty-two radiology residents were randomized into two groups of 11 residents each receiving five identical musculoskeletal (MSK) radiology lectures. One group received lectures through ARS and the other through traditional didactics. A pretest and identical posttest were completed by all residents at baseline and eight months later, respectively. Residents also completed a pre and post five-question Likert scale survey designed to measure perceptions of ARS.

Results

Wilcoxon rank sum tests revealed no statistically significant difference between the two groups of residents on the pretest (p = 0.47) or the posttest (p = 0.41). Of the five questions designed to gauge perceptions of ARS, “How often do you study radiology outside of work?” resulted in statistical significance between groups after the lecture series via ordinal logistic regression, with the ARS group six times more likely to study compared to the non-ARS group (Odds ratio = 6.52, P = 0.04, 95% Confidence Interval [1.1, 38.2]). There was no statistical difference in response to this question prior to the lecture series.

Discussion

Use of ARS was associated with increased likelihood of studying radiology without significant difference in long-term learning outcomes.

## Introduction

Radiology residency programs are increasingly adopting audience response systems (ARS) such as Nearpod, Poll Everywhere, and Radiological Society of North America (RSNA) Diagnosis Live as a means to promote active learning in hopes of greater retention of clinically relevant information. In fact, the aforementioned technologies are the three most commonly used ARS in the United States radiology residency programs today, with RSNA Diagnosis Live quickly becoming the most widely used technology with over 100 participating institutions [1].

No consensus currently exists as to the most effective form of education for radiology residency programs. Because more residency programs are relying on these newer innovative techniques in educating the future generation of diagnostic radiologists, it is imperative that these specific tools are studied, analyzed, and investigated meticulously as to whether they aid in retention of information for learners. Furthermore, the effect of ARS on learning outcomes remains somewhat ambiguous [2-10]. There remains conflicting evidence as to the effectiveness of ARS with regards to short-term and long-term learning retention [2-5]. For example, Schackow et al. have shown that ARS are associated with improved learning outcomes and short-term retention of learning in family medicine residents [2], but Robson et al. [3] demonstrated that ARS did not lead to increased short-term retention of learning amongst dental students when compared to similar dental students receiving traditional lectures without ARS. Furthermore, Atlantis and Cheema [5] concluded in a systematic review that there was an absence of quality evidence on the effectiveness of ARS technologies for improving learning outcomes in healthcare professionals. No study to date has reliably shown improved long-term learning outcomes for healthcare professionals with the use of an ARS, to the best of our knowledge.

The primary objective of this study was to assess the effect of ARS on long-term learning outcomes amongst radiology residents at a single institution. Residents were randomized into two learning groups, one group receiving ARS (RSNA Diagnosis Live) for five lectures throughout the year, and another group receiving five lectures with the exact same content but without ARS. Our hypothesis was that those residents randomized to the group receiving ARS would have improved long-term learning outcomes when compared to the group of residents not receiving ARS technology.

## Materials and methods

Study population

Institutional Review Board (IRB) approval was obtained for this randomized unblinded prospective pilot study, and written informed consent in accordance with the Declaration of Helsinki was obtained from all participants in the study. The study sample consisted of all 22 radiology residents at our institution. Residents were randomized into one of two groups, with 11 residents randomized to a group that would receive RSNA Diagnosis Live for five musculoskeletal (MSK) radiology lectures throughout the 2017-2018 academic year, and 11 residents randomized to a group that would receive an identical MSK radiology lecture without RSNA Diagnosis Live. RSNA Diagnosis Live was chosen as the platform for the ARS used in this study because it was readily available at no cost to our institution. Randomization was stratified based on postgraduate year level of training to ensure equal number of participants in each group based on learning levels. The mean age in our study sample was 30 years (range: 27-39 years), with 15 males and seven females. The mean age of males was 30 years (range: 27-39 years), while the mean age of females was 30 years (range: 28-35 years).

Study design

In July of 2017, all residents completed a 50-item multiple choice question pretest via pencil and paper, the content of which was based on the five MSK lectures to be delivered through the upcoming academic year. Ten questions were administered from each of the five MSK lectures, five of which were image-based and the other five were text questions without images. Between September and February of 2017-2018, five identical MSK lectures were given to both groups of residents, with the intervention group of 11 residents receiving the lecture through RSNA Diagnosis Live, and the control group of 11 residents receiving the identical lecture without RSNA Diagnosis Live. The topics of the five lectures were acetabular fractures, pelvic trauma, shoulder replacement, imaging of anterior cruciate ligament repair, and musculoskeletal infection. To help minimize some investigator bias (since all three MSK faculty members were co-authors of this study), two of the five MSK lectures were delivered by one MSK faculty member, two additional lectures were delivered by a different MSK faculty member, and the final MSK lecture was delivered by a third MSK faculty member. On the day of the lectures, the order of the lecture to be delivered to either the intervention or control arm of participants was randomly selected to balance possible presenter fatigue during the second lecture. Each lecture was 45 minutes in duration, and the identical lectures with and without ARS were given immediately after the other during the 12:00 PM to 1:30 PM hours. Conditions for the lectures, such as room location, lecturer, and lighting, etc. were nearly identical for both lectures during a given day with the exception of the use of ARS for the intervention group. Makeup lectures were scheduled by the chief residents for participants that missed lectures to ensure 100% attendance for all five lectures, which was achieved. Makeup lectures were given by the same faculty member that delivered the original lecture. Finally, a 50-item multiple choice question posttest that was identical to the pretest was administered and completed by all participants at the end of March 2018 via paper and pencil.

Evaluation of outcomes

**Figure 1:**
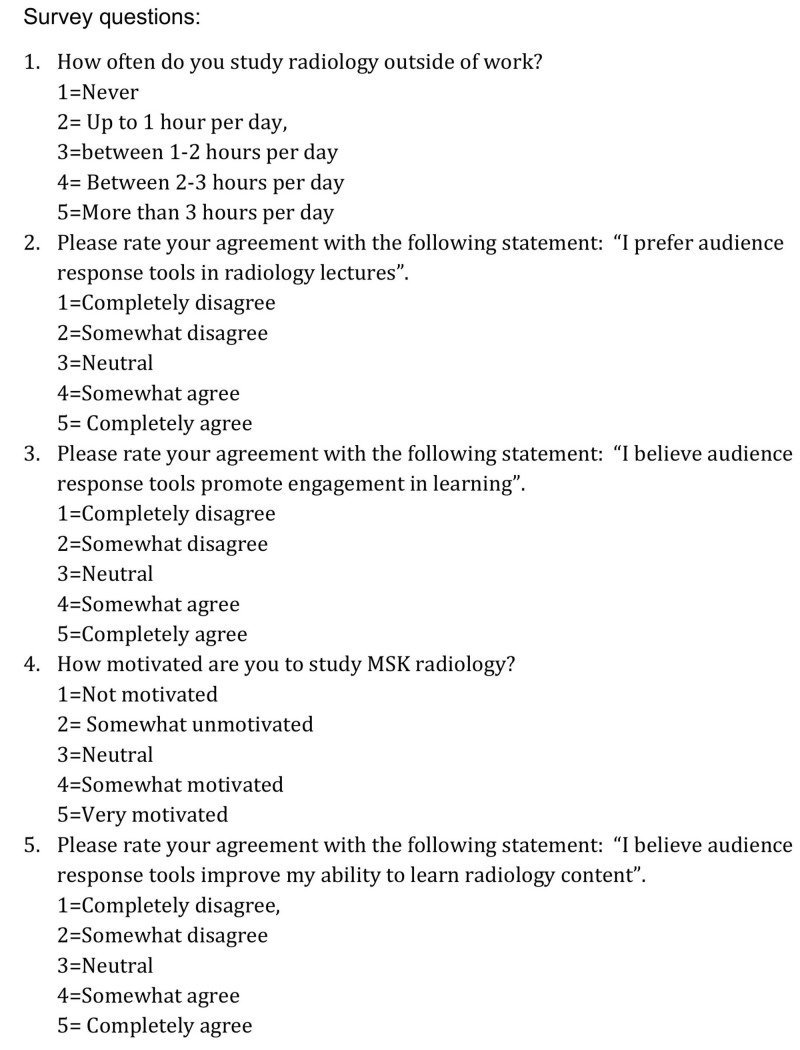
Survey. Survey questions distributed to all residents at the beginning and end of the study. Survey responses were based on a five-point Likert scale as shown above.

The primary outcome of interest was long-term retention of information, gauged by scores on the posttest, given eight months after the pretest. Several secondary outcomes were also assessed. Improvement between pretest and posttest was measured by the difference in scores between the two tests given eight months apart. In addition, scores from the musculoskeletal section of the American College of Radiology (ACR) In-Service examination were compared a priori between the intervention and control arm of the study to assess if there was any correlation between the educational intervention of ARS examined in this study with success on a national standardized examination. Finally, perceptions and motivations regarding studying and learning were assessed via an identical survey (Figure 1) administered at the beginning (July 2017) and end of the study (March 2018). The survey was administered electronically via Qualtrics (Provo, UT) software anonymously to all residents by a mentor of the primary investigator on this manuscript who had no relation to any of the residents in this study. The investigators in this study did not have access to individual responses to the survey, but only anonymized data from the survey.

Statistical analysis

All statistical analyses were carried out by Stata 14.1 statistical software (College Station, TX). Because there lacked a normal distribution of scores on the pretest and posttest, a nonparametric test was used to assess the effect of ARS on pre- and posttest scores. Thus, a Wilcoxon rank sum test was used to evaluate our primary outcome, to ascertain if there was a statistically significant difference (p < .05) in posttest scores between the intervention arm and control arm of our study. A Wilcoxon rank sum test was used to assess if there was improvement in scores between pre and posttest amongst the intervention and control arm of our study. The Wilcoxon rank sum test was used to assess if there was any statistically significant difference between ACR In-Service exam scores within the MSK section between the intervention and control arms of our study, since there failed to be a normal distribution of scores. In addition, because responses in our survey were assessed via a Likert scale, ordinal logistic regression was employed to evaluate differences in responses to the questions between the intervention and control groups in our study.

## Results

Despite the small sample size in our study, baseline characteristics were balanced amongst the intervention and control arm of our study (Table 1), such as mean age, gender, and median pretest scores. Results from the Wilcoxon rank sum tests revealed no statistically significant difference in pretest scores (p = 0.47), posttest scores (p = 0.41), improvement in scores between posttest and pretest (p = 0.60), or the MSK section of the ACR In-Service exam (p = 0.53) between those that received ARS for the five lectures and those that did not receive ARS. Thus, the use of an ARS did not correlate with improved long-term learning outcomes in our study.

**Table 1:** Baseline characteristics of study sample.

	Intervention Group (N = 11 Residents)	Control Group (N = 11 Residents)
Mean Age	30.0	30.1
Sex		
Male	7 (63.6%)	8 (72.7%)
Female	4 (36.4%)	3 (27.3%)
Median Pretest Score	52%	48%

However, residents that received ARS did perform better than those that did not receive ARS on the posttest and the MSK ACR In-Service exam, but this was not statistically significant (Table 2). For example, residents’ median posttest score in the intervention arm of the study was eight points higher than residents in the control arm of the study. Similarly, median scores on the MSK ACR In-Service exam were eight points higher for those that received ARS compared to those that did not. Median improvement between posttest and pretest was also four points higher for the residents that received ARS.

**Table 2:** Distribution of mean and median test scores by study sample, intervention group, and control group. * Musculoskeletal ^†^ American College of Radiology

	Study Sample (N = 22 Residents)	Intervention Group (N = 11 Residents)	Control Group (N = 11 Residents)
Mean score on pretest	47.8	48.2	47.5
Median score on pretest	50	52	48
Mean score on posttest	66.8	68.4	65.3
Median score on posttest	64	70	62
Mean difference in scores between posttest and pretest	19	20.2	17.8
Median difference in scores between pretest and posttest	18	22	18
Mean scaled score on MSK^*^ ACR^†^ In-Service exam	63.6	66.1	61.1
Median scaled score on MSK^*^ ACR^†^ In-Service exam	63	67	59

At baseline, our survey results showed no statistically significant difference between the two arms of our study on any of the five dimensions/questions measured, a finding expected with successful randomization (Table 3). However, at the end of the study, residents that received audience response had 6.52 (Odds Ratio = 6.52, P = 0.04, 95% Confidence Interval [1.1, 38.2]) times the odds of studying radiology more outside of work when compared to residents that did not receive ARS (Question 1), and this was statistically significant (Table 3). The other dimensions (Questions 2-5) were not statistically different between the two arms of our study at the completion of the study.

**Table 3:** Survey response analysis at the beginning of study and end of study.

	Odds Ratio	P-value	95% Confidence Interval	Likelihood-Ratio Test for Proportionality of Odds (P-value)
Baseline				
Question 1	0.42	0.31	0.08-2.24	0.36
Question 2	0.22	0.08	0.04-1.20	0.74
Question 3	0.97	0.97	0.19-4.94	0.24
Question 4	1.49	0.63	0.29-7.58	0.06
Question 5	1.51	0.61	0.31-7.34	0.27
End of Study				
Question 1	6.52	0.04	1.11-38.18	0.39
Question 2	0.12	0.08	0.01-1.25	0.6
Question 3	0.25	0.27	0.02-2.92	0.61
Question 4	1.69	0.51	0.35-8.24	0.08
Question 5	0.33	0.21	0.06-1.86	0.46

Finally, median responses to the survey for the entire study sample were assessed. By the end of the study, median responses of all residents for questions 2,4, and 5 demonstrated that residents preferred ARS in radiology lectures (Table 4). In addition, these results showed that residents believed ARS promoted engagement in learning and helped to improve their ability to learn radiology content.

**Table 4:** Median survey responses at baseline and end of study.

	Median Response for Entire Study Sample (N = 22 Residents)
Baseline	
Question 1	I study radiology up to 1 hour per day outside of work
Question 2	I somewhat agree to the statement, “I prefer audience response tools in radiology lectures”
Question 3	I completely agree to the statement, “I believe audience response tools promote engagement in learning”
Question 4	I am somewhat motivated to study MSK radiology
Question 5	I somewhat agree to the statement, “I believe audience response tools improve my ability to learn radiology content”
End of Study	
Question 1	I study radiology 1 to 2 hours per day outside of work
Question 2	I completely agree to the statement, “I prefer audience response tools in radiology lectures”
Question 3	I completely agree to the statement, “I believe audience response tools promote engagement in learning”
Question 4	I am somewhat motivated to study MSK radiology
Question 5	I completely agree to the statement, “I believe audience response tools improve my ability to learn radiology content”

## Discussion

There remains a paucity of evidence in the literature assessing long-term learning outcomes in medical professionals via ARS. Our study did not show improved long-term learning outcomes in radiology residents amongst any dimension measured, including a posttest testing content from five different lectures, or the MSK section of a national standardized exam, the ACR In-Service exam. The results from the survey, however, demonstrated a statistically significant likelihood of studying radiology more outside work amongst the residents that did receive ARS. In addition, by the end of the study, median responses to the survey showed the strong preference for ARS in radiology lectures as well as the strong belief that ARS may help promote engagement as well as ability to learn radiology content.

Despite the fact that our initial hypothesis was not corroborated with the data in this study, our study does suggest a role for using ARS in didactic and case-based radiology lectures in educating the next generation of radiology residents. As educators continue to search for ways to optimally teach millennials, an approach that incorporates technology into education, such as RSNA Diagnosis Live, has been shown to cater to this “technologically native” demographic. The benefits of ARS include increased engagement during lectures without the pressure of being in a “hot seat” as has been done in traditional case-based radiology conferences, as well as the possibility of motivating residents to study more radiology content, as our study showed. Learner preference remains an important educational principle of andragogy, and radiology residents at our institution preferred ARS in their lectures, based on survey results. Furthermore, the motivation through self-directed learning cultivated through ARS is of paramount importance in radiology education since radiology residents often have to learn outside of the reading room in order to master the large breadth of knowledge necessary to pass core exams and become a competent radiologist.

The results of our study are supported by other similar studies in the literature. De Oliveira-Santos et al. showed no statistically significant difference in final exam scores between students that received ARS for four oral and maxillofacial radiology undergraduate lectures when compared to students that did not receive ARS [11]. However, this same study also demonstrated that students strongly felt that ARS positively influenced their performance. Our study revealed similar findings with a smaller sample size of 22 residents, compared to the 74 students in de Oliveira-Santos’ study.

Shah et al. demonstrated review quizzes delivered by ARS correlated with in-training exam scores in emergency medicine residents and was viewed positively [12]. Our study did not specifically evaluate whether ARS correlated with ACR In-Service exam scores, but rather tested whether those that received ARS performed better on the MSK section of the ACR In-Service exam score. Based on Shah et al.’s study, perhaps there is a role of ARS in helping prepare residents for in-training exams.

Some ARS now have the ability to track resident performance through granular data. For example, RSNA Diagnosis Live, the ARS used in this study, has a tagging feature whereby questions can be tagged under headings such as MSK or neuroradiology based on the content of the question. In addition, RSNA Diagnosis Live automatically aggregates data on every individual’s correct and incorrect responses to questions presented in a lecture. Thus, resident performance can be tracked with time. A future study could examine whether deficiencies in trainee learning could be improved by tailoring different RSNA Diagnosis Live games to a specific learner based on content that a trainee has yet to master. This future study could shed light on whether RSNA Diagnosis Live specifically improves learning outcomes via granular data inherent in its technologic software. It should be noted that the results from one of our dedicated lectures on acetabular fractures have been published in a separate study [13].

Our study has several limitations. First, we had a small sample size of 22 residents with 11 residents in each randomization assignment, thus limiting the power of our study. In addition, results from our study cannot necessarily be generalized to residents in other programs, as our study represents one study at one institution, with five lectures covering only one subspecialty in radiology. Finally, we could not control for participant and even observer bias in our study protocol. Due to informed consent policy, all residents were aware of their randomization assignment, and this may have influenced performance on tests or responses to the survey. In addition, the MSK lecturers were all well aware of the study design, and knowledge of the design could have inadvertently biased the lecturers when teaching radiology content to the two different groups of residents. However, having multiple MSK lecturers for the duration of our study should have theoretically decreased some observer bias. Finally, although in theory randomization was successful based on results from Table 1, the authors could not control for confounding variables such as exposure to MSK radiology during rotations that may have biased the results of the study. However, due to randomization, we would expect this effect to be balanced amongst the two arms of the study. More studies are needed involving larger sample sizes, with multiple institutions, and testing radiology content from more than one subspecialty to fully evaluate the effect of ARS on long-term learning outcomes in radiology residents.

## Conclusions

Use of ARS was not associated with improved long-term learning outcomes in our study. Despite this, there does appear to be a role for using ARS in radiology lectures in radiology residency programs. Residents firmly believe that ARS promotes engagement, helps learn radiology content and improves their ability to learn.
